# Inhibition of transketolase by oxythiamine altered dynamics of protein signals in pancreatic cancer cells

**DOI:** 10.1186/2162-3619-2-18

**Published:** 2013-07-27

**Authors:** Jiarui Wang, Xuemei Zhang, Danjun Ma, Wai-Nang Paul Lee, Jing Xiao, Yingchun Zhao, Vay Liang Go, Qi Wang, Yun Yen, Robert Recker, Gary Guishan Xiao

**Affiliations:** 1 Genomics & Functional Proteomics Laboratories, Osteoporosis Research Center, Creighton University Medical Center, 601 N 30th ST, Suite 6730, Omaha, NE 68131, USA; 2 Metabolomics Core, UCLA Center of Excellence in Pancreatic Diseases, Harbor-UCLA Medical Center, Torrance, CA 90502, USA; 3 Department of Respiratory Medicine, Dalian Medical University, Dalian 116027, China; 4 Molecular Clinical Pharmacology, City of Hope Cancer Center, Duarte, CA 90101, USA; 5 Department of Respiratory Medicine, The Fifth Hospital of Dalian, Dalian 116027, China; 6 The Medical College of Dalian University, Dalian Economic & Technological Development Zone, Dalian 116622, China

**Keywords:** Quantitative proteomics, Pancreas cancer, ^15^ N stable isotope, Phosphorylation, Turnover rate, Metabolic inhibitor, Metabolic therapy, Transketolase, Oxythiamine

## Abstract

Oxythiamine (OT), an analogue of anti-metabolite, can suppress the nonoxidative synthesis of ribose and induce cell apoptosis by causing a G1 phase arrest *in vitro* and *in vivo*. However, the molecular mechanism remains unclear yet. In the present study, a quantitative proteomic analysis using the modified SILAC method (mSILAC) was performed to determine the effect of metabolic inhibition on dynamic changes of protein expression in MIA PaCa-2 cancer cells treated with OT at various doses (0 μM, 5 μM, 50 μM and 500 μM) and time points (0 h, 12 h and 48 h). A total of 52 differential proteins in MIA PaCa-2 cells treated with OT were identified, including 14 phosphorylated proteins. Based on the dynamic expression pattern, these proteins were categorized in three clusters, straight down-regulation (cluster 1, 37% of total proteins), upright “V” shape expression pattern (cluster 2, 47.8% total), and downright “V” shape pattern (cluster 3, 15.2% total). Among them, Annexin A1 expression was significantly down-regulated by OT treatment in time-dependent manner, while no change of this protein was observed in OT dose-dependent fashion. Pathway analysis suggested that inhibition of transketolase resulted in changes of multiple cellular signaling pathways associated with cell apoptosis. The temporal expression patterns of proteins revealed that OT altered dynamics of protein expression in time-dependent fashion by suppressing phosphor kinase expression, resulting in cancer cell apoptosis. Results from this study suggest that interference of single metabolic enzyme activity altered multiple cellular signaling pathways.

## Introduction

It has been known for decades that most tumor cells and tissues enhanced glucose metabolism by glycolysis [1,2]. Although its causal relationship with cancer cell proliferation is still unclear, the phenomenon has been developed a reliable technique for detecting and classifying tumors by fluorodeoxyglucose positron emission tomography (FDG-PET) [3,4]. In recent years, this metabolic alteration of malignant cells has been observed in multiple cancer cells, and it has become an important aspect for design of anticancer drugs that inhibits glycolysis and other relevant metabolic processes. Several small molecules, including 2-deoxyglucose, lonidamine, 3-bromopyruvate, imatinib and oxythiamine (OT), have shown the effectiveness in anticancer activity in vitro and in vivo [5-16]. They are currently in the clinical and pre-clinical phase. Some other compounds also exhibit potential anticancer activity by modulating glucose metabolism [16].

OT is a thiamine antagonist and inhibits transketolase (TK) which is an enzyme of the pentose phosphate pathway in animals. As transketolase reaction plays a vital role of the pentose phosphate pathway, inhibition of transketolase will suppress the pentose phosphate pathway and interrupt the synthesis of these important coenzymes ATP, CoA, NAD(P)+, FAD, and genetic material, RNA and DNA in cancer cells. OT can suppress the nonoxidative synthesis of ribose and cause cell apoptosis by inducing a G1 phase arrest *in vitro* and *in vivo*[14,15,17,18]. Although the exactly molecular mechanism is not clear, it has been accepted that the decreased biological macromolecular synthesis can inhibit cell proliferation and induces cell apoptosis. Therefore, these features of metabolism are actually used for cancer therapeutic approach known as “metabolic therapy” [19,20].

In the present study, a dynamic proteomic method was adapted to analyze the effects of antimetabolite OT on dynamic changes of protein expression in pancreatic cancer cells, thus to understand the molecular mechanism underlying antimetabolite interference.

## Materials and methods

### Chemicals and regents

^15^ N enriched algal amino acid mixture (^15^ N enrichment, 98%) was purchased from Cambridge Isotope Laboratory Inc. (Andover, MA). Fetal bovine serum (FBS) was purchased from Irvine Scientific (Santa Ana, CA). Dulbecco’s modified Eagles’s medium and antimycotic were from Gibco (Calsbad, CA). Sequence grade trypsin solution was from Promega (Madison, WI).

Acetonitrile was purchased from Thermo Fisher Scientific (Rockford, IL). Materials employed for gel electrophoresis were purchased from BioRad. Water was prepared using a Milli-Q system (Millipore, Bedford, MA). Other chemicals employed were purchased from Sigma (St. Louis, MO). This project was approved by Creighton University Institutional Review Board.

### *In vitro* cytotoxic activity

The cell cytotoxicity of OT against the MIA PaCa-2 cells was determined by MTT assay [21,22]. The cells at exponential phase were dispensed in 96-well plates at a density of 1 × 10^4^ cells per well. The cells were stimulated with different concentrations of OT for 2 days. The cells were then incubated in 20 μl MTT (3-(4, 5-dimethylthiazol-2-yl)-2, 5-diphenyl tetrazolium bromide) (Sigma, USA) in growth medium at 37°C for 4 h lysed in 100 μl of dimethyl sulfoxide (Sigma, USA) for 10 min. The absorbance in each well was measured at 490 nm by an ELx800 Absorbance Microplate Reader (Biotek, CA). The cell viability and IC_50_ value were calculated by the following equations: cell viability = mean optical density of experimental group/mean of the control × 100%; IC_50_ value = concentration of OT at 50% cell viability.

### Cell culture

Human pancreatic carcinoma cell line MIA PaCa-2 was maintained in MEM supplemented with 10% fetal bovine serum and 1% antibiotic antimycotic at 37°C in 5% CO2 until 80% confluence when the experiment started [23,24]. Experiments were set up in two groups: dose- and time-dependent groups. For the dose-dependent group, the cells were stimulated with 5, 50 and 500 μM OT for 48 hours, respectively. The unstimulated cells were considered as control. For the time-dependent group, the cells were stimulated with 50 μM OT in MEM containing natural amino acids or 50% of ^15^ N algal amino acid mixture (^15^ N enrichment, 98%) for 12 and 48 h. The unstimulated cells were considered as the zero time point. Each treatment was repeated four times with 10 mL/flask. The cell pellets were then collected for further analysis.

### Protein sample preparation

The cell pellets were immediately washed three times with ice-cold PBS. Cells were harvested in 2-DE lysis buffer with protease inhibitor set III and phosphatase inhibitor set II (Calbiochem, La Jolla, CA). The suspension was sonicated at 100 Watt for 3 × 5 s and centrifuged at 20,000 × g for 30 min. Protein concentration was measured by Bradford assay using bovine serum albumin as the standard. The samples were stored at -80°C until analysis.

### Two-Dimensional Gel Electrophoresis (2-DE)

Two-DE was performed as previously described [23,24]. Briefly, five hundred micrograms of proteins were mixed with a rehydration solution (Bio-Rad, Hercules, CA) containing 7 M urea, 2 M thiourea, 4% CHAPS, 50 mM DTT, 0.2% biolyte 3–10, 0.1% biolyte 4–6, and 0.1% biolyte 5–8 and a trace of bromophenol blue to a total volume of 300 μL. The mixtures were pipetted into IPG strip holder channels. After 14 h of rehydration, the strips, pH 3–10 NL, were transferred to the isoelectric focusing (IEF) holders (Bio-Rad, Hercules, CA). Prefocusing and focusing were performed on the IPGphor platfor (Bio-Rad, Hercules, CA) (500 V hold 2.5 h, linear 500–1000 V increase 1 h, 1000 V hold 1 h, linear 1000-8000 V increase 1.5 h, and 8000 V hold 60,000 KV h). Following IEF separation, the gel strips were equilibrated twice for 15 min each with equilibration buffer I and II (37.5 mM Tris-Cl, pH 8.8, 20% glycerol, 2% SDS, 6 M urea, with 2% DTT in buffer I and 2.5% iodoacetamide in buffer II, respectively). The equilibrated gel strips were then placed onto 8-16% Tris–HCl gel, and sealed with 0.5% agarose in a Protean Plus Dodeca cell (Bio-Rad, Hercules, CA) until the bromophenol blue reached the bottom of the gels.

After 2-DE, the gels were stained with Pro-Q Diamond [25,26]. Then the gels were stained using SYPRO-Ruby (Molecular Probes, Eugene, OR) or visualized with the Coomassie Brilliant Blue R-250 (Merck, Germany) overnight at room temperature. Following 2-DE and protein staining, stained gels were scanned with a Pharox FX molecular imager (Bio-Rad) with a 532 nm laser excitation and a 580 nm band-pass emission filter. Spot detection, quantification and matching were identified using PDQuest 8.0 software (Bio-Rad). The intensity of each protein spot was normalized to the entire gel intensity of all spots detected. Quantitative analysis was performed using the Student’s t-test. The confidence level was 95%. Only those proteins of intensity difference > 2-fold change were selected for MALDI-TOF/TOF MS.

### In-gel Trypsin digestion

Protein spots of interest were excised from the gels and in-gel digested with trypsin as previously described [27]. Briefly, gel pieces were destained with 100 mM ammonium bicarbonate in 30% ACN and dried in a vacuum centrifuge. Ten ng of modified trypsin (Promega, Madison, WI) in 25 mM ammonium bicarbonate was added, followed by incubation 20 h at 37°C. The supernatant was collected, and then the peptides were further extracted three times from the gel pieces with 0.1% trifluoroacetic acid (TFA), 60% ACN with vortexing for 45 min at room temperature. Peptides extracts were vacuum-dried.

### MALDI-TOF-MS

For mass spectrometric analysis, the peptides extracts were brought up in 10 μL of 0.1% TFA and cleaned using C18 ZipTip (Millippore, MA). Typically, 2 μL of a-cyano-4-hydroxycinnamic acid (HCCA) matrix in 50% ACN/0.1% TFA was used to elute peptide onto the ground steel plate (Bruker, Germany). The internal standard from Bruker Bruker (MH1: angiotensin II, 1046.5420 Da; angiotensin I, 1296.6853 Da; substance P, 1347.7361 Da; bombesin, 1619.823 Da; ACTH clip 18–39, 2465.199 Da) were used for mass scale calibration. The resulting peptides were extracted and analyzed by MALDI TOF/TOF mass spectrometer (Ultraflex III, Bruker, Germany) in the reflector mode and for sequence analysis in the “lift” mode.

### Protein identification and spectral data analysis

The MS/MS spectrum from MALDI measurements were then searched against the Mus musculus subset (16235 sequences) of UniProt KB/Swiss-Prot/TrEMBL database (database version 57.15; 515203 sequences) using the Mascot v 2.2 search program (Matrix Science, London, United Kingdom) (http://www.matrixscience.com). Search parameters for the database search with Mascot were set as follows: enzyme, trypsin; allowance of up to one missed cleavage peptide; fixed modification parameter, carbamidomethylation (C); variable modification parameters, oxidation (at Met); mass tolerance for precursor ions was ±1.2 Da; mass tolerance for fragment ions, ±0.6 Da.

Mascot scores of proteins or peptides were used for protein identification (p < 0.05). In the case of peptides matching to multiple members of a protein family, the positive identified protein was selected based on both the highest score and the highest number of matching peptides. These peaks were externally calibrated with peptide standards from Bruker (MH1: angiotensin II, 1046.5420 Da; angiotensin I, 1296.6853 Da; substance P, 1347.7361 Da; bombesin, 1619.823 Da; ACTH clip 18–39, 2465.199 Da).

The synthesis rates of the differential proteins were calculated according to our in-house algorithms [24,25]. One-way ANOVA with the Tukey’s adjustment was used for multiple comparisons in SPSS 13.0 (SPSS Inc., Chicago, IL).

### Pathway analysis

Ingenuity Pathway Analysis (IPA) (Ingenuity Systems, Inc., Redwood City, CA, http://www.ingenuity.com) was used for pathway, network and functional analyses of differential proteins in the present study.

### *K*-means clustering

Protein ratios were transformed to the log scale (base 2) before clustering [28]. The Cluster 3.0 freeware software package was used for clustering analysis (http://bonsai.hgc.jp/~mdehoon/software/cluster/software.htm). Repeated (10–100) K-means clustering of proteins was based on Pearson correlation coefficient of their expression profiles [29].

### Western blotting analysis

Western blotting analysis was performed as described previously [29]. Briefly, after SDS-PAGE separation, proteins were then transferred to PVDF membranes (Millipore, CA) according to the manufacture’s protocol, and antibody labeling was visualized using ECL reagent (Pierce Biotech Inc., Rockford, IL). Western blot score was a fraction of β-actin or β-tubulin, and measured in Quantity One (Bio-Rad).

To examine whether Annexin A1 is expressed in pancreatic cancer, we separately tested a set of serum samples from patients (pancreatic tumor; pT2/pT3) and healthy volunteer subjects with age-matched. The patient serum samples (n = 7) and the healthy volunteer blood samples (n = 12) were collected by City of Hope National Medical Center and NCI-designated Cancer Center (Duarte, CA) with proper informed consent according to a protocol approved by the Institute Review Board. All samples used in this study were further approved by the Institutional Review Board at Creighton University. The blood samples in BD Vacutainer® Blood Collection Tubes (BD Ventures, L.L.C., NJ) were fractionated by centrifuging at 1,000 × g for 10 min. The serum samples were immediately divided into aliquots and frozen at -80°C. The mean (±SD) age for the tumor patients was 61.3 (±8.1) years, and for the healthy volunteer group, 60.3 (±5.4) years. We measured serum levels of Annexin A1 by using Western blotting analysis. All the experiments were performed in triplicates.

## Results

### Oxythiamine caused protein expression in a dose-dependent manner

**Figure 1:**
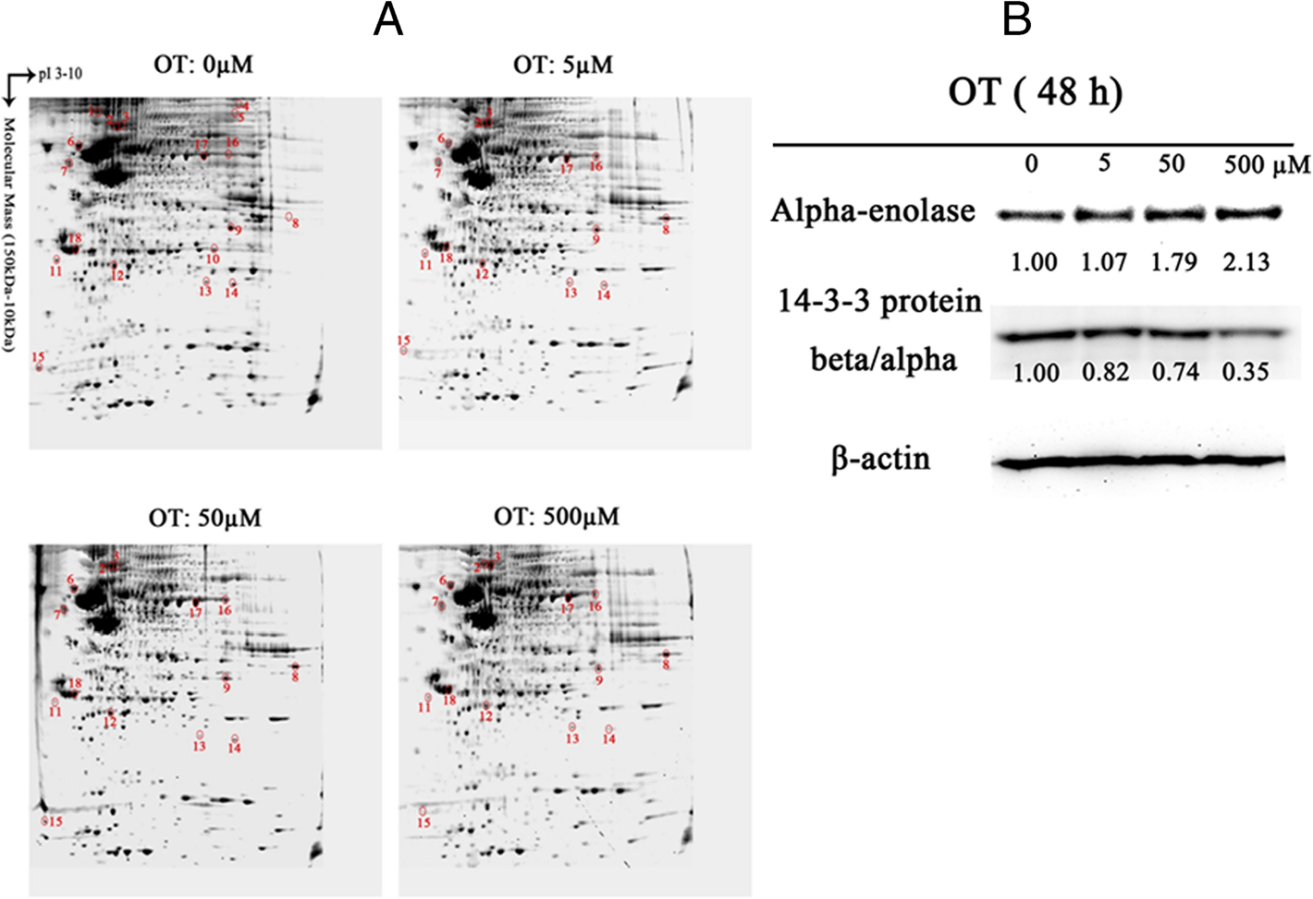
**Dose-dependent analysis of the MIA PaCa-2 cells to oxythiamine. A.** Two-DE patterns of whole-cell proteins obtained from MIA PaCa-2-2 cells treated with oxythiamine (0 μM, 5 μM, 50 μM, and 500 μM). On the basis of PDQuest software analysis, ratios of normalized spot intensities of four different concentrations of oxythiamine were calculated, and spots showing more than 2.0-fold difference with statistical significance (p < 0.05) were selected after these 2D gels were stained by SYPRO Ruby. **B.** The validation of two differential proteins (alpha-enolase and 14-3-3 protein beta/alpha) for dose–dependent group. Western blot scores are annotated under the bands. The results were well consistent with the 2-DE analyses.

Using MTT assay, we determined the toxicity of OT to MIA PaCa-2 cells and found that the IC50 of OT for MIA PaCa-2 is 14.95 μM (Additional file 1: Figure S1). To study whether OT caused protein expression in a dose-dependent fashion, MIA PaCa-2 cells were treated with the stepwise concentrations of OT (0 μM, 5 μM, 50 μM and 500 μM). Protein expression in MIA PaCa-2 cells was profiled using two-dimensional gel electrophoresis (2DE) (Figure 1A). From Figure 1A, we found that OT altered protein expression in a dose dependent manner. The differentially expressed proteins were selected using criterion of > 2-fold difference among groups with statistical significance (p < 0.05), and led to identification of eighteen proteins (Table 1). Among them, fourteen proteins were suppressed significantly, and four were induced remarkably, by OT treatment. Interestingly, heat shock cognate 71 kDa protein was detected and identified from two adjacent spots in the gel (spots #2 and #3 in Figure 1A), suggesting that this protein may be underwent post-translational modification by OT treatment.

**Table 1:** Identified proteins of differential expression of the MIA PaCa-2 cells in the OT-dose dependent group by MALDI-TOF/TOF MS

**Spot No.**^**a**^	**Accession number**^**b**^	**Protein name**	**Ratio of OT-induced differential expression**^**c**^			
			**0**	**5**	**50**	**500 (μM)**
1	P14625	Endoplasmin	1	0.27	0.33	0.20
2	P11142	Heat shock cognate 71 kDa protein	1	0.41	025	0.19
3	P11142	Heat shock cognate 71 kDa protein	1	3.13	2.58	2.79
4	P13639	Elongation factor 2	1	0.13	0.09	0.21
5	P08238	Heat shock protein HSP 90-beta	1	0.26	0.18	0.14
6	P07237	Protein disulfide-isomerase	1	0.07	0.22	0.09
7	P13489	Ribonuclease inhibitor	1	0.17	0.24	0.19
8	P22626	Heterogeneous nuclear ribonucleoproteins A2/B1	1	7.51	11.45	10.96
9	P63244	Guanine nucleotide-binding protein subunit beta-2-like 1	1	0.65	0.43	0.38
10	Q15056	Eukaryotic translation initiation factor 4H	1	0.32	0.15	0.27
11	P56537	Eukaryotic translation initiation factor 6	1	0.40	0.24	0.17
12	P09211	Glutathione S-transferase P	1	0.61	0.57	0.26
13	P30086	Phosphatidylethanolamine-binding protein 1	1	0.63	0.29	0.3
14	P62826	GTP-binding nuclear protein Ran	1	0.37	0.42	0.15
15	P60660	Myosin light polypeptide 6	1	0.34	0.49	0.08
16	P51854	Transketolase	1	4.17	3.30	1.85
17	Q6GMP2	Alpha-enolase	1	0.89	2.13	2.29
18	P31946	14-3-3 protein beta/alpha	1	0.76	0.50	0.55

a. The numbers of the identified protein spots are indicated in Figures 1, 2 and 3;

b. Swiss-Prot/TrEMBL primary accession number.

c. The ratio of expression level (intensity on the gel) of each protein spot were calculated based on the control (0 μM OT) group.

To further verify the expression patterns of these proteins in MIA cells, we selected alpha-enolase and 14-3-3 protein beta/alpha to examine protein expression by Western blot (Figure 1B). The level of alpha-enolase was increased by OT treatment, while expression of 14-3-3 protein beta/alpha was suppressed by OT at a stratified dose (Figure 1C). The results were in agreement with the 2-DE analyses.

### Oxythiamine altered dynamics of protein expression in MIA PaCa-2 cells

To investigate whether OT treatment caused dynamic changes of cellular protein expression in MIA PaCa-2 cells, we treated MIA cells with OT at dose of 50 μM in different time points (0 h, 12 h and 48 h). To detect functional cellular protein signals in MIA cells in response to OT treatment, we used ^15^ N labeled amino acids as tracers to culture the cells, and dynamic synthesis rate of total proteins newly synthesized was calculated [24] (Figure 2A). Clearly, OT caused dynamic changes of total protein expression in time dependent fashion. A total of 46 proteins were identified (Table 2).

**Figure 2:**
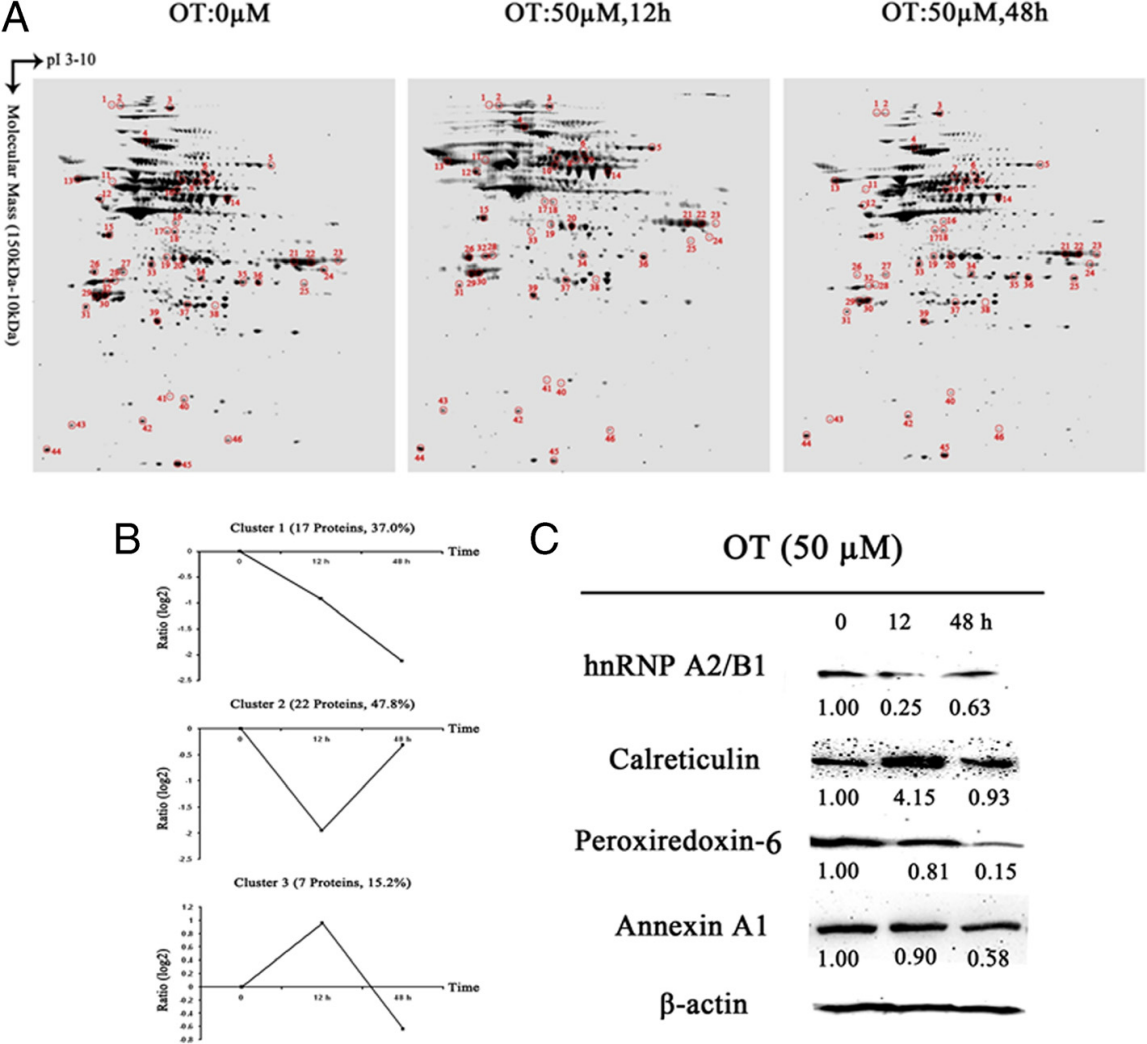
**Time-dependent analysis of the MIA PaCa-2 cells to oxythiamine. A.** Two-DE patterns of total proteins from the MIA PaCa-2 cells treated by OT with 0, 12 and 48 h. On the basis of PDQuest software analysis, ratios of normalized spot intensities of four different concentrations of oxythiamine were calculated, and spots showing more than 2.0-fold difference with statistical significance (p < 0.05) were selected after these 2D gels were stained with SYPRO Ruby. **B.** The temporal profile patterns of 45 differential proteins. The 0 min value was used as a basal time point to calculate the protein fold changes in 12 h and 48 h. After transformed to the logarithmic scale (base 2), the temporal changed profiles of 45 differentially proteins were algorithmically subdivided into nine clusters using K-means clustering method. The protein number of each cluster is indicated. **C.** The validation of four differential proteins (peroxiredoxin-6, annexin A1, calreticulin and heterogeneous nuclear ribonucleoproteins A2/B1) for the time-dependent group. Western blot scores are annotated under the bands. The results were well consistent with the 2-DE analyses.

**Table 2:** Identified proteins of differential expression of the MIA PaCa-2 cells in the OT-time dependent group by MALDI-TOF/TOF MS

**Spot No.**^**a**^	**Accession number**^**b**^	**Protein name**	**Ratio of OT-induced differential expression**^**c**^					
			**Total proteins**			**Phosphorylated proteins**		
			**0**	**24**	**48 (h)**	**0**	**24**	**48 (h)**
1	P49321^e^	Nuclear autoantigenic sperm protein	1.00	0.37	1.35	1.00	0.09	0.05
2	P14625^f^	Endoplasmin	1.00	1.17	0.26	--	--	--
3	Q14697 ^e^	Neutral alpha-glucosidase AB	1.00	0.41	0.89	--	--	--
4	P11142^d^	Heat shock cognate 71 kDa protein	1.00	0.62	0.45	1.00	0.13	0.08
5	P29401 ^f^	Transketolase	1.00	3.23	0.77	--	--	--
6	P31948 ^d^	Stress-induced-phosphoprotein 1	1.00	0.61	0.28	1.00	1.17	0.19
7	P30101 ^d^	Protein disulfide-isomerase A3	1.00	0.30	0.06	--	--	--
8	P17987 ^d^	T-complex protein 1 subunit alpha	1.00	0.39	0.21	--	--	--
9	P31146 ^e^	Coronin-1A	1.00	0.45	0.74	--	--	--
10	P31943 ^d^	Heterogeneous nuclear ribonucleoprotein H	1.00	0.88	0.49	--	--	--
11	P07237 ^e^	Protein disulfide-isomerase	1.00	0.37	0.84	--	--	--
12	P13489 ^d^	Ribonuclease inhibitor	1.00	0.91	0.23	--	--	--
13	P27797 ^f^	Calreticulin	1.00	3.58	0.95	--	--	--
14	P06733 ^f^	Alpha-enolase	1.00	2.37	0.82	1.00	0.24	0.07
15	Q86VC0 ^f^	40S ribosomal protein SA	1.00	1.35	2.00	--	--	--
16	P52597 ^e^	Heterogeneous nuclear ribonucleoprotein F	1.00	0.31	0.73	--	--	--
17	P52907 ^e^	F-actin-capping protein subunit alpha-1	1.00	0.49	0.81	--	--	--
18	O75821 ^e^	Eukaryotic translation initiation factor 3 subunit I	1.00	0.29	0.72	1.00	0.73	0.29
19	P47755 ^e^	F-actin-capping protein subunit alpha-2	1.00	0.43	0.87	1.00	0.20	0.03
20	P04083 ^d^	Annexin A1	1.00	0.87	0.48	--	--	--
21	P04406 ^e^	Glyceraldehyde-3-phosphate dehydrogenase	1.00	0.39	1.25	--	--	--
22	P04406 ^e^	Glyceraldehyde-3-phosphate dehydrogenase	1.00	0.41	1.32	--	--	--
23	P09651 ^e^	Heterogeneous nuclear ribonucleoprotein A1	1.00	0.17	1.39	--	--	--
24	P22626 ^e^	Heterogeneous nuclear ribonucleoproteins A2/B1	1.00	0.06	0.33	--	--	--
25	P52895 ^e^	Aldo-keto reductase family 1 member C2	1.00	0.48	4.02	--	--	--
26	Q9BYE2 ^d^	Serine/threonine-protein kinase 13	1.00	0.11	0.07	--	--	--
27	P08758 ^d^	Annexin A5	1.00	0.43	0.36	--	--	--
28	Q9Y275 ^d^	14-3-3 protein epsilon	1.00	0.34	0.12	1.00	0.36	0.15
29	P31946 ^d^	14-3-3 protein beta/alpha	1.00	0.39	0.22	1.00	0.31	0.74
30	P63104 ^d^	14-3-3 protein zeta/delta	1.00	0.42	0.28	1.00	0.87	0.14
31	P56537 ^e^	Eukaryotic translation initiation factor 6	1.00	0.39	0.47	1.00	0.76	0.41
32	P62258 ^f^	Tumor necrosis factor ligand superfamily member 13B	1.00	2.13	0.29	1.00	0.33	0.17
33	Q6ZU15 ^e^	Septin-14	1.00	0.04	0.78	--	--	--
34	Q13162 ^e^	Peroxiredoxin-4	1.00	0.20	0.53	--	--	--
35	P18669 ^e^	Phosphoglycerate mutase 1	1.00	0.08	2.59	--	--	--
36	P63244 ^e^	Guanine nucleotide-binding protein subunit beta-2-like 1	1.00	0.49	0.91	--	--	--
37	P04792 ^d^	Heat shock protein beta-1	1.00	0.43	0.28	--	--	--
38	P30041 ^d^	Peroxiredoxin-6	1.00	0.89	0.37	--	--	--
39	P09211 ^d^	Glutathione S-transferase P	1.00	0.77	0.42	1.00	0.26	0.04
40	P16949 ^e^	Stathmin	1.00	0.35	0.46	--	--	--
41	P15531 ^d^	Nucleoside diphosphate kinase A	1.00	0.97	0.09	--	--	--
42	P32119 ^e^	Peroxiredoxin-2	1.00	0.37	0.49	--	--	--
43	P60660 ^d^	Myosin light polypeptide 6	1.00	0.88	0.23	--	--	--
44	Q8N1F1^f^	Putative uncharacterized protein NCRNA00188	1.00	1.15	0.50	--	--	--
45	P05109 ^e^	Protein S100-A8	1.00	0.29	0.73	--	--	--
46	P62937 ^e^	Peptidyl-prolyl cis-trans isomerase A	1.00	0.07	0.19	--	--	--
47	Q969J3	Loss of heterozygosity 12 chromosomal region 1 protein	--	--	--	1.00	0.13	0.02
48	P63241	Eukaryotic translation initiation factor 5A-1	--	--	--	1.00	0.14	0.05

a. The numbers of the identified protein spots are indicated in Figures 1, 2, and 3;

b. Swiss-Prot/TrEMBL primary accession number.

c. The ratio of expression level (intensity on the gel) of each protein spot were calculated based on the control (0 μM OT) group.

d. Protein expression in Cluster 1.

e. Protein expression in Cluster 2.

f. Protein expression in Cluster 3.

On the basis of the time course study, the temporal expression patterns of OT-induced proteins were analyzed. Forty-five proteins identified from forty-six protein spots, which showed a 2-fold or greater change, revealed three different profile patterns, straight down-regulation (cluster 1, 37% of total proteins), upright “V-shape” expression pattern (cluster 2, 47.8% total), and downright “V-shape” pattern (cluster 3, 15.2% total) (Figure 2B).

To further verify the expression patterns of these proteins in time dependent manner, four proteins were selected for further analyses by western blot (Figure 2C). The expression of peroxiredoxin-6 and annexin A1 in cluster 1 were decreased upon the OT treatment. Calreticulin in cluster 3 was increased significantly at the 12 h time point, but significantly decreased to its basal level at the 48 h time point. Heterogeneous nuclear ribonucleoproteins A2/B1 in cluster 2 showed an opposite trend to calreticulin (Figure 2C).

### OT suppressed phosphoprotein expression in MIA PaCa-2 cells

Particularly, reversible phosphorylation of proteins plays a significant role that occurs in both prokaryotic and eukaryotic organisms, including cell apoptosis and differentiation, signal transduction, cell-cycle progression, energy storage and utilization [30-33]. To study whether cellular phosphor proteins were also altered by OT treatment, we analyzed phosphor protein expression in MIA cells (Figure 3). Obviously, OT suppressed expression of cellular phosphor proteins significantly. A total of 14 phosphorylated proteins were identified (Figure 3), and listed in Table 2. Of these 14 phosphorylated proteins, 6 proteins in Cluster 1 (heat shock cognate 71 kDa protein, 14-3-3 protein epsilon, 14-3-3 protein beta/alpha, 14-3-3 protein zeta/delta, eukaryotic translation initiation factor 6 and glutathione S-transferase P) showed concordant changes with total proteins, 6 proteins of Clusters 2 and 3 (nuclear autoantigenic sperm protein, stress-induced-phosphoprotein 1, alpha-enolase, eukaryotic translation initiation factor 3 subunit I, F-actin-capping protein subunit alpha-2 and tumor necrosis factor ligand superfamily member 13B) were discordant, and 2 (loss of heterozygosity 12 chromosomal region 1 protein and eukaryotic translation initiation factor 5A-1) were only observed in the phosphorylated patterns (Figure 3, Table 2). Decrease in phosphorylated proteins induced by OT treatment indicated that the inhibition of pentose phosphate pathway might cause a time-dependent decrease of phosphorylated proteins patterns.

**Figure 3:**
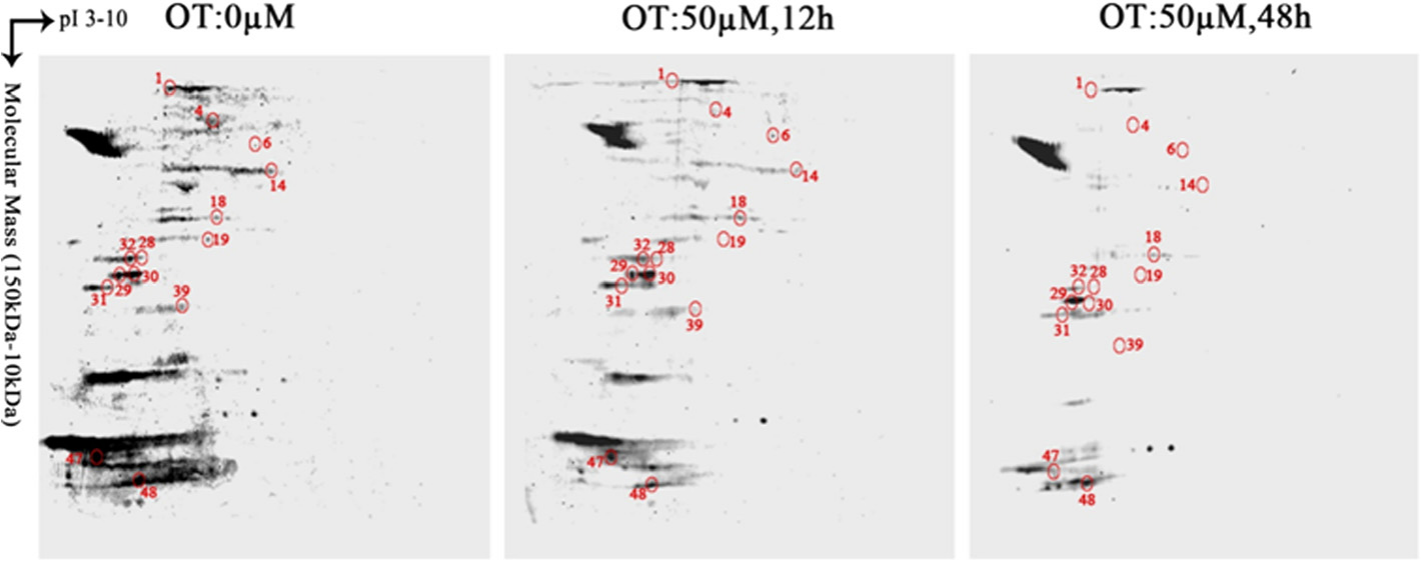
Two-DE patterns of phosphorylated proteins from the MIA PaCa-2 cells treated by OT with 0, 12 and 48 h.

### Functional annotation of the differential proteins identified

**Figure 4:**
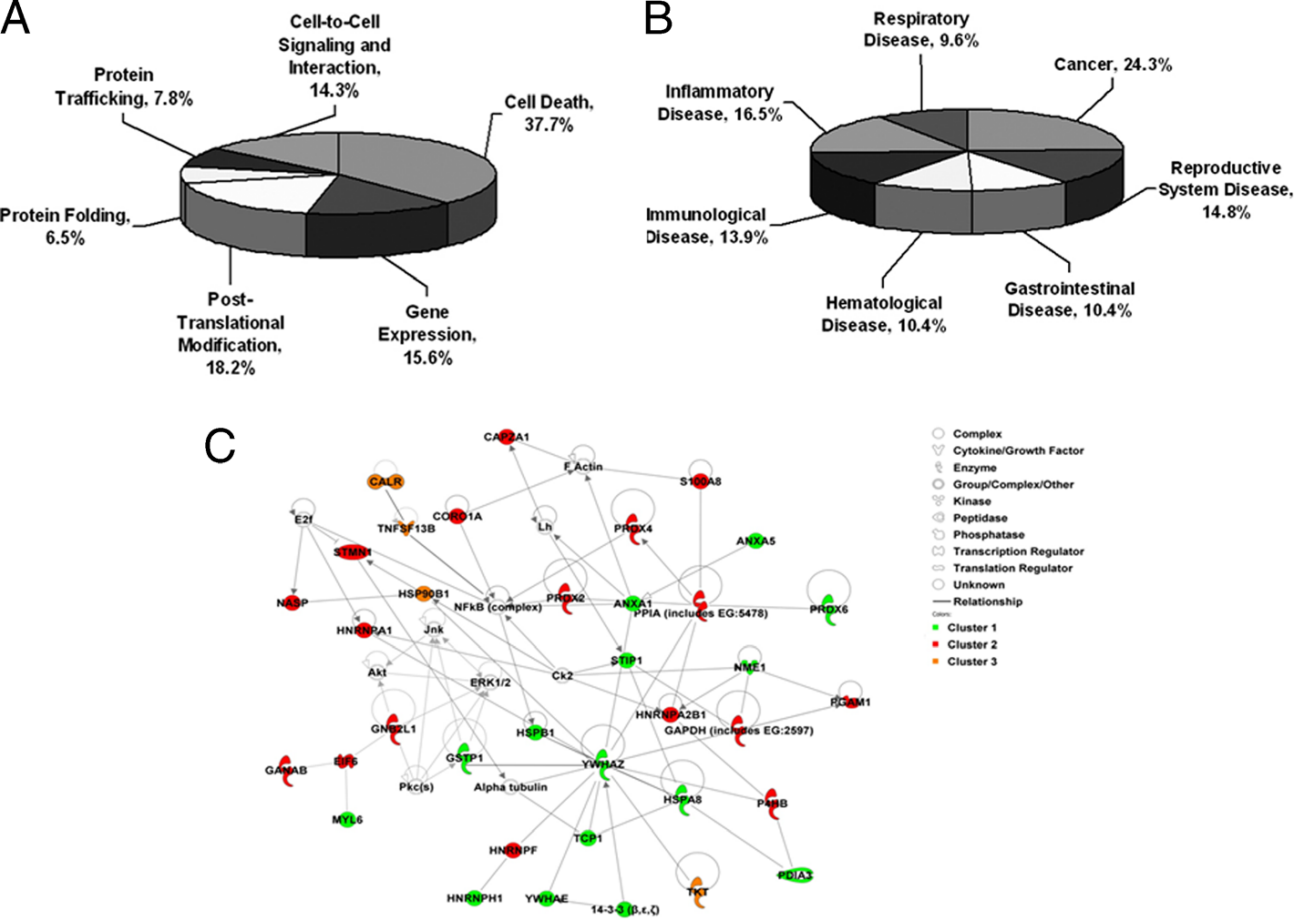
**Functional characteristics of the differential proteins identified. A.** Functional analysis of fifty-two differential proteins via Ingenuity database (http://www.ingenuity.com). Molecular functional annotation including cell death (29, 37.7%), gene expression (12, 15.6%), post-translational modification (14, 18.2%), cell-to-cell signaling and interaction (11, 14.3%), protein folding (5, 6.5%), protein trafficking (6, 7.8%). **B.** Classification related with diseases and disorders including cancer (28, 24.3%), reproductive system disease (17, 14.8%), gastrointestinal disease (12, 10.4%), hematological disease (12, 10.4%), immunological disease (16, 13.9%), inflammatory disease (19, 16.5%), respiratory disease (11, 9.6%). **C.** The analysis of network based on the Ingenuity database. A total of 36 differential proteins, which showed three different profiles response to OT treatment, were matched in the signaling network of cancer and cell death.

To annotate the proteins identified in this study, all 52 differential proteins were analyzed with Ingenuity Pathway Analysis (IPA) (http://www.ingenuity.com). The potential functional annotation of these proteins revealed that most of the proteins were involved in signaling transduction of cell death, including cell death signaling (29, 37.7%), gene expression (12, 15.6%), post-translational modification (14, 18.2%), cell-to-cell interaction (11, 14.3%), protein folding (5, 6.5%), and protein trafficking (6, 7.8%) (Figure 4A). Moreover, many proteins were involved in multiple signaling pathways that play role in incidence of diseases, including cancer (28, 24.3%), reproductive system disease (17, 14.8%), gastrointestinal disease (12, 10.4%), hematological disease (12, 10.4%), immunological disease (16, 13.9%), inflammatory disease (19, 16.5%), respiratory disease (11, 9.6%) (Figure 4B).

The proteins quantitatively measured with turnover rate (a total of 36 proteins) were also annotated using IPA bioinformatics (Figure 4C). The differentially expressed proteins in three clusters are involved in multiple signaling pathways that are associated with tumor cell survival, and apoptosis. For examples, 14-3-3 proteins (14-3-3 protein epsilon, 14-3-3 protein beta/alpha and 14-3-3 protein zeta/delta) in cluster 1, play roles in cell survival, cell proliferation, anti-apoptosis and anti-tumor suppression by activating ERK/MAPK signaling pathway and the mitochondrial apoptotic machinery [34-37]. Peroxiredoxin-2 and peroxiredoxin-4 in cluster 2, which were up-regulated in many cancers [38-40], showed a significant decrease upon OT-treatment for 12 hr but increased to almost basal level after OT treatment for 48 hr. It suggested that these proteins in cluster 2 might be the early response molecules upon OT-treatment. Calreticulin in cluster 3, which is associated with proimmunogenic killing in cancer cells [41], was up-regulated upon OT-treatment for 12 hr but then down-regulated to almost basal level after treatment for 48 hr, suggesting that OT-induced cell apoptosis might be associated with proimmunogenic killing in the early time.

### OT interrupted the protein synthesis rate

Dynamic protein synthesis, which is the results of protein synthesis and degradation, is the key to regulate the cell signaling and determine the cell destiny [42,43]. In our study, dynamic protein synthesis rates of differential proteins were able to be determined by our recently developed method [24]. A total of 41 proteins were measured, including 7 proteins with a turnover rate < 45% (protein disulfide-isomerase A3, alpha-enolase, tumor necrosis factor ligand superfamily member 13B, eukaryotic translation initiation factor 6, nucleoside diphosphate kinase A, myosin light polypeptide 6 and phosphoglycerate mutase 1), 5 proteins with a turnover rate > 65% (heterogeneous nuclear ribonucleoproteins A2/B1, peroxiredoxin-2, peroxiredoxin-6, endoplasmin and nuclear autoantigenic sperm protein) and other 29 proteins with a turnover rate between 45% and 65% (Table 3). Proteins with high protein turnover rate indicated they may be actively involved in some cell physiological processes, especially in drug treatment cells.

**Table 3:** OT interrupted the protein synthesis rates

**Protein name**	**Protein synthesis rate**		
	**48 (**^**15 **^**N only)**^g^	**50 μM OT (h)**	
		**12**^h^	**48**^i^
Endoplasmin	71%	--^j^	42%
Heat shock cognate 71 kDa protein	65%	--	52%
Ribonuclease inhibitor	59%	--	36%
Annexin A1	55%	--	37%
Glyceraldehyde-3-phosphate dehydrogenase	61%	--	70%
Heterogeneous nuclear ribonucleoprotein F	63%	--	78%
Heterogeneous nuclear ribonucleoproteins A2/B1	67%	--	45%
Peroxiredoxin-4	65%	--	73%
Peroxiredoxin-6	70%	--	52%

g. Protein turnover rates were measured from cells treated with ^15^ N enriched medium for 48 h without OT treatment.

h. Protein turnover rates were measured from cells treated with ^15^ N enriched medium and 50 μM OT for 12 h.

i. Protein turnover rates were measured from cells treated with ^15^ N enriched medium and 50 μM OT for 48 h.

j. “--” means no significant changes of protein turnover rates compare to basal group (0 hr).

We also examined the time-dependent relationship of protein synthesis to OT treatment in MIA PaCa cells (Table 3). Because of the low protein concentration recovered from the 2-D gel, we were only able to determine the fraction of new synthesis in eight proteins at the various time points of OT treatment. Table 3 shows the time response of the fraction of new synthesis (% new synthesis in 12 and 48 hours) of eight proteins. There were no significant mass shifts of the peptide spectra at 12 h time point of OT treatment. It indicated that there may be no the new syntheses of proteins before 12 h. Fraction of new syntheses of five proteins were decreased, three were increased at 48 h time point of OT treatment. It suggested the differential effects of OT treatment on protein turnover of the eight proteins.

**Figure 5:**
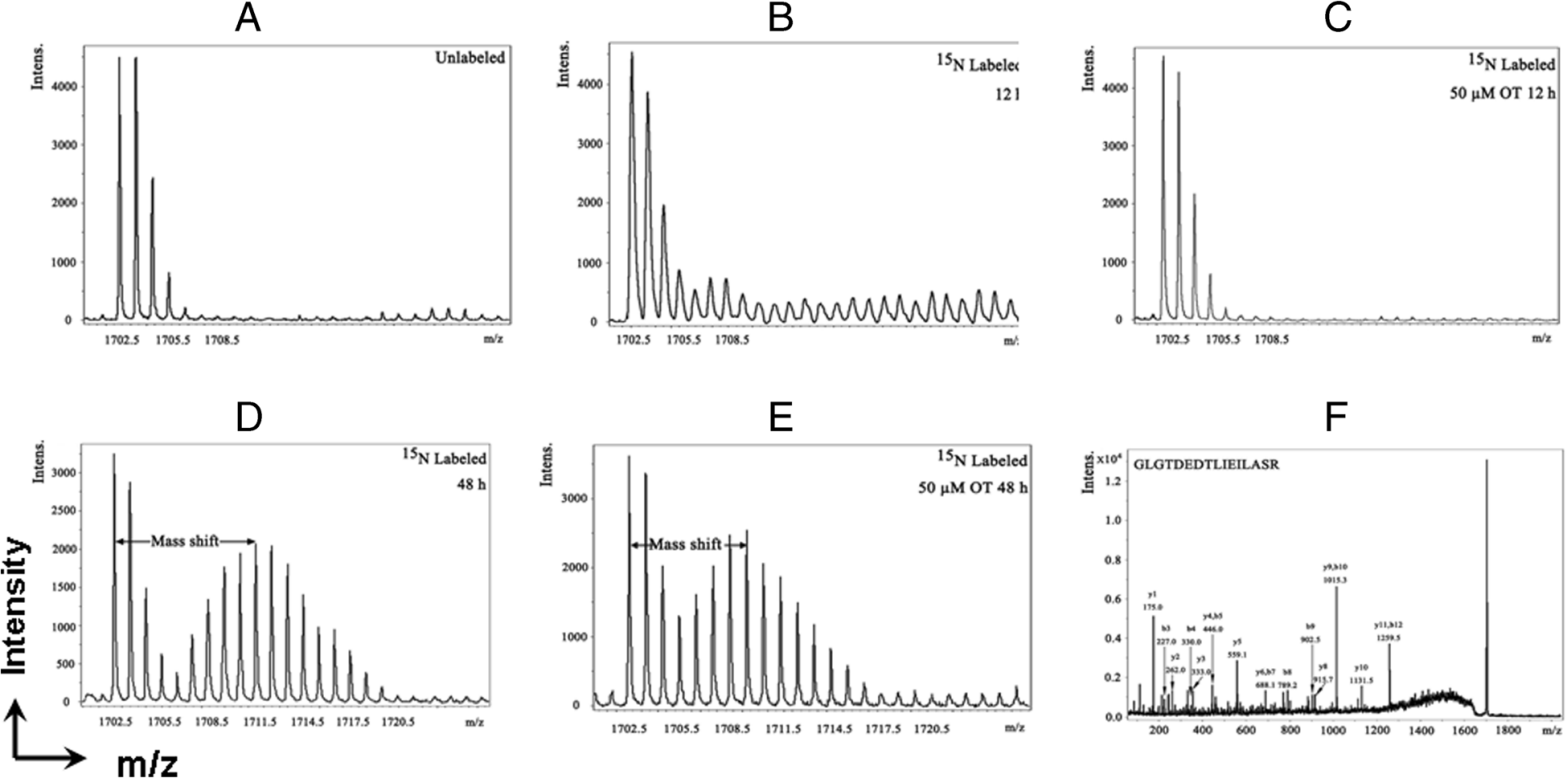
**OT interrupted protein synthesis rate of annexin A1.** MS spectra of 1702.5 m/z fragment from spot #20 of lysates of cells grown in the presence of natural amino acids **(A)**, 50% ^15^ N enriched medium and 0 μM OT for 12 h **(B)** and 50% ^15^ N enriched medium and 50 μM OT for 12 h **(C)**, 50% ^15^ N enriched medium and 0 μM OT for 48 h **(D)**, ^15^ N enriched medium and 50 μM OT for 48 h **(E)**. The synthesis rate of a protein was based on the isotopomer distribution of these spectra. **(F).** shows the MS/MS spectrum of this fragment in the “lift” mode. Sequences were confirmed from the labeled b- and y-ions in the spectrum using a Mascot database search.

The effect of ^15^ N incorporation on the isotopomer distribution of a peptide from protein spot #20 is illustrated in Figure 5. The isotopomer distribution of fragment 1702.5 m/z in spot #20 (Annexin A1) is shown in Figure 5A-F. The distribution of the unlabeled fragment (^14^ N) is showed in Figure 5A.

The same peptide, labeled with ^15^ N enriched medium and treated with or without 50 μM OT for 12 h, is illustrated in Figure 5B,C. There were no significant differences between the three spectra. It indicated protein synthesis was not interrupted at the 12 h time point of OT treatment in the MIA PaCa-2 cells. Figure 5D shows the spectrum from cells grown in 50% ^15^ N enriched medium for 48 h. It suggested the obvious spectrum shift in mass comparing with ^14^ N labeled spectrum. The spectrum in Figure 5E is from the cells grown in ^15^ N enriched medium and 50 μM OT for 48 h, which showed smaller mass shift than that of only 50% ^15^ N enrichment. Turnover rates were then calculated by multiple linear regression analysis of the observed peptide spectrum. The fraction of new synthesis of the peptide was reduced from 55% to 37% by OT treatment. Using a Mascot database search, we determined that the sequence of the peptide is part of a protein annexin A1 (Figure 5F). It suggested that OT could inhibit the synthesis of annexin A1.

### Expression of Annexin A1 in human pancreatic cancer

**Figure 6:**
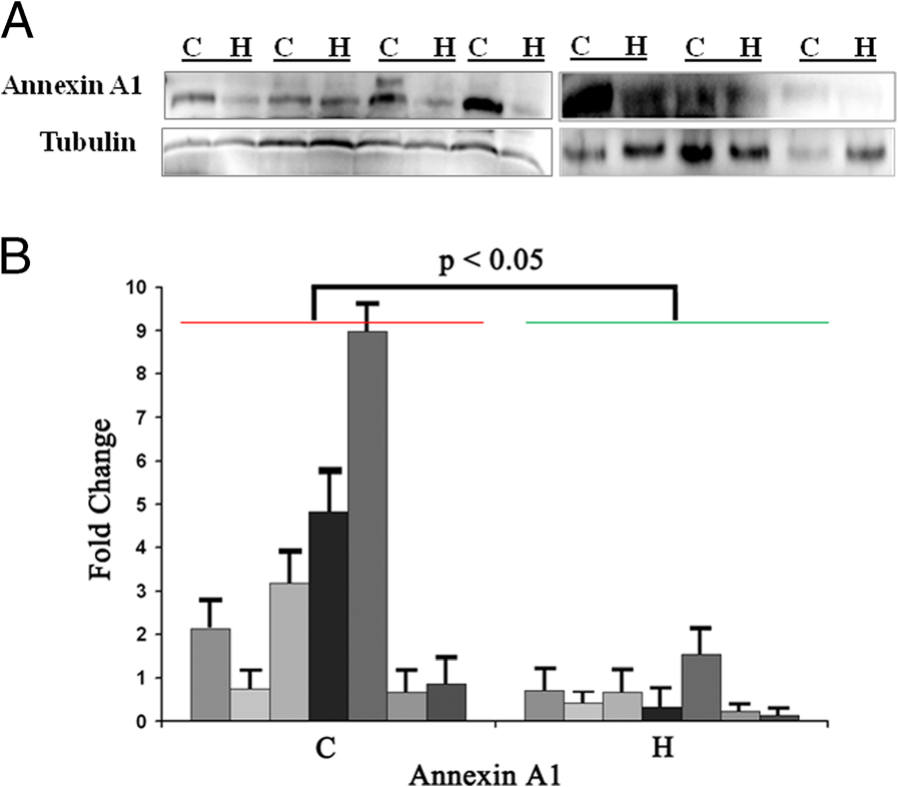
**Differantial expression of annexin A1 in pancreatic cancer and healthy plasma. A.** Western blot of seven random pairs of human plasma from pancreatic cancer and healthy people. **B.** Statistical analysis of the Western blot in the Panel **A**. Band intensity was normalized to that of tubulin. *C*, plasma from pancreatic cancer patiant; *H*, palsma from healthy people.

Annexin A1, a major substrate for epidermal growth factor receptor kinase, plays an important role in cancer development and progression [44,45]. Although expression of Annexin A1 was reported to be associated with a number of cancers including pancreatic cancer [46], the molecular mechanism underlying is unknown. To further validate the expression of Annexin A1 in patients with pancreatic cancer, Western blot analysis was performed in archived clinic plasma samples from patients who had pancreatic cancer (n = 7) and healthy control (n = 7) (Figure 6A/B). Clearly, Annexin A1 is expressed significantly in pancreatic cancer patients compared to the healthy controls (p < 0.05) (Figure 6B). These results agreed well with our *in vitro* study above, suggesting that Annexin A1 may be developed as a surrogated marker potentially useful for early detection of pancreatic cancer.

## Discussion

Cancer cells utilize glucose maximally as a main source of energy supply and substrates for proliferation through glycolytic metabolic pathways [1,2]. Inhibition of the activity of the key enzymes (e.g., transketolase/transadolase) in these metabolic networks, resulting in significant limitation of glucose utilization, provides an ideal strategy for an effective therapy of cancer. A number of our previous studies have shown that inhibition of activity of either transketolase in the pentose phosphate cycle, or glycogen phosphorylase causes cell cycle arrest leading to cancer cell apoptosis [17,23,47,48]. In this study, we found that transketolase inhibitor OT altered dynamics of cellular protein expression in MIA PaCa-2 cells by interrupting the rates of protein *de novo* synthesis. This study provides 1) an important clinical implication for identifying novel cellular protein signals/targets that are associated mechanistically with cancer treatment; 2) a novel approach for detecting signal molecules that initiate drug resistance.

Small molecule antimetabolites are among the more effective chemotherapeutic agents in use today. Currently, gemcitabine, 5-fluorouracil (5-FU), and imatinib, are commonly used for the treatment of pancreatic cancer [49-51]. However, the response rate to either gemcitabine, or imatinib, and patient survival, are poor [52,53]. There is an urgent need to discover additional chemotherapeutic targets such as metabolic enzymes that play a crucial role in controlling the growth of cancer cells. In this study, we found that OT caused protein expression in a time dependent fashion. Peroxiredoxin-6 of cluster 1, which can suppress TRAIL-mediated cell death in human cancer cells by binding to death effector domain caspase [54], was constantly down-regulated by the duration of OT treatment (from 0 hr to 48 hrs) (Figure 2B). It implicated that OT induced cell death by hperoxiredoxin-6 related TRAIL-induced pathway. However, peroxiredoxin-2 and peroxiredoxin-4 of cluster 2, the same ubiquitous family of peroxiredoxin-6, which were up-regulated in many cancers [38-40], were shown in an upright “V” shape (Figure 2B). It suggested that the two proteins might be the only early response molecules upon OT-treatment comparing with peroxiredoxin-6. Calreticulin of cluster 3 was shown in a downright “**V**” shape (Figure 2B). It suggested calreticulin might have an opposite function of OT-induced cell apoptosis as the early time response molecular comparing with peroxiredoxin-2 and peroxiredoxin-4. The protein expression pattern in three clusters suggested that metabolic dynamic changes (dynamic changes of transketolase activity) in MIA cancer cells in response to OT treatment caused dynamic changes of cellular protein signals. Protein expression pattern indicates that dynamics of these protein expressions differs in MIA cells in response to OT treatment. Interestingly, expression of these proteins in cluster 2 and 3 was no significant difference in MIA cells treated with OT for 48 hrs, but significantly changed in MIA cells treated with OT for 12 hrs, compared to that in MIA cells at basal line (0 hrs). Early response proteins (e.g., Glyceraldehyde-3-phosphate dehydrogenase, S100A8) in clusters 2 and 3 may play an important role in MIA cells against OT treatment. Several studies revealed that expression of both Glyceraldehyde-3-phosphate dehydrogenase and S100A8 are suppressed in Raw264.7 cells [25,26,55]or bone cells [56,57] in response to toxins or tobacco smoke, suggesting that these proteins play an important role in cell defense (survival). Suppression of the protein expression may be part of cell emergent response mechanism since OT treatment cut off supplies of substrates and energy for cancer cell proliferation.

Because of a wild range of protein concentration in cells, it is the most difficult to study in a truly comprehensive manner. Standard proteomics usually compares amounts of proteins in cells in two different states (e.g. disease vs. normal) or conditions (e.g. treatment vs. non treatment) [58]; it does not address the dynamics of the proteome in the different biological states that are being compared, nor does it provide information about the mechanisms whereby the system changes from one state to the other. Thus, data obtained from this study may provide unique cell survival mechanism.

Previous study showed that dynamic changes of metabolic enzyme activity determined the metabolic sensitivity of cancer cells to the treatment [47], therefore, the early responsive protein signals upon OT treatment may be indicatives for the sensitivity of pancreatic cancer cells to the treatment in molecular level.

The dynamic changes of the cellular molecules (mRNAs, proteins, and metabolites) depend upon the physiological, developmental, or pathological state of living cells [59]. A change in the proteome may be the most important outcomes of a cellular response, such as autophagy, to exogenous stimuli. Autophagy is a constitutive, catabolic process leading to the lysosomal degradation of cytosolic proteins and organelles. Dynamic changes of proteins identified in Clusters 3 and 4 may reflect the cellular autophaging phenomena.

Because of a wild range of protein concentration in cells, it is the most difficult to study changes of protein expression in a truly comprehensive manner. Standard proteomics usually compares amounts of proteins in cells in two different states (e.g. disease *vs.* normal) or conditions (e.g. treatment *vs.* non treatment) [58]; it does not address the dynamics of the proteome in the different biological states that are being compared, nor does it provide information about the mechanisms whereby the system changes from one state to the other. This study provides more dynamic information of cellular protein signals than our previous studies and others [23], which some dynamic information of proteins in clusters 2 and 3 may be missed (or so-called “false negative” errors) when standard proteomics approach is used in our previous studies.

Protein turnover is the balance between protein synthesis and protein degradation (or breakdown), which is believed to decrease with age in all senescence organisms including humans. This results in an increase in the amount of damaged protein within the body. It is unknown if this is a cause or consequence of aging but it seems likely that it is in fact both. The damaged protein results in a slower protein turnover which then results in more damaged protein causing an exponential increase in damage to all protein within the body and to aging. Protein turnover is being considered as a missing dimension in proteomics for biomedical research [59]. The dynamics of protein turnover is one of key features to the understanding of regulation of protein expression and protein-protein interaction in cells [24,60]. The level of expression of a protein depends on the rates of its synthesis and degradation. Thus the turnover of a protein is an important indicator of its functional significance in cells. Despite its evident importance, the role of protein turnover has not previously been considered in analyses of the proteome. Protein turnover can be quantified on a protein-by-protein basis. With the established method [24], in this study we were able to quantitatively measure the rates of newly synthesized proteins. Among 41 proteins measured, 7 proteins are with a turnover rate of < 45%, 5 proteins are with a turnover rate of > 65%, and 29 proteins are with a turnover rate between 45% and 65%. The turnover rates of the proteins with extreme high or low levels are related to specific status of cell physiology (e.g., suppression of metabolic signaling).

Intriguingly, we did not observe *de novo* synthesized peptides in MIA cells treated with OT in 12 h. This is reasonable since it takes more than 24 hr for a specific gene translating to the cognate protein. The explanation for detection of the differential expressed proteins at 12-h treatment may be 1) because OT directly or indirectly activated pathways of degradation resulting in rapid degradation of cellular functional proteins; 2) because OT directly or indirectly turned on pathways of posttranslational modification leading to the increased amount of proteins. This notion can be demonstrated from Figure 6B/C, obviously, *de novo* synthesis of proteins cannot be detected.

Previous study revealed that some active metabolic pathways, TCA, glycolysis, oxidative phosphorylation and the pentose phosphate pathway, were interconnected with the critical signaling pathways in proliferating cells [61]. Inhibiting of these metabolic pathways could affect the biological macromolecular synthesis and suppress cell proliferation [23,47]. In previous studies it had been shown OT could cause the inhibition of nucleic acid synthesis metabolic through increasing imbalances in pentose phosphate cycle [18]. In our study, the expression profile patterns of cellular phosphorylated proteins of MIA PaCa-2 were significantly inhibited by OT treatment (Figure 3). This phenomenon might be caused by inhibition of biological macromolecular synthesis or some critical signaling pathways related phosphorylation.

Certainly, this is a principal proof study examining whether interference of the interactive metabolic and cell signaling pathways alters expression of protein signals associated with cellular abnormal activity of protein turnover. Although information obtained in a single pancreatic cancer cell line MIA PaCa-2 may be limited to a study in other cell lines, protein signals identified in this study may provide useful information for further development of novel biomarkers and/or drug targets for administration of pancreatic cancer.

## Conclusion

This study revealed that inhibition of single metabolic enzyme transketolase by OT altered dynamics of cellular protein expression by interfering rates of *de novo* protein synthesis in MIA PaCa-2 pancreatic cancer cells. These cellular dynamic protein signals may be involved in several signaling pathways of cell apoptosis. These results may help understand better molecular mechanism of the anti-tumor activity of OT.

## Abbreviations

2-DE: Two-dimensional electrophoresis; MALDI: Matrix assisted laser desorption ionization; TOF/TOF MS: Time-of-flight/time-of-flight mass spectrometry; PMF: Peptide mass fingerprinting; OT: Oxythiamine; TK: Transketolase.

## Competing interests

The authors declare that they have no competing interests.

## Authors’ contributions

JW and DM, carried out rat experiments. DM, drafted the manuscript. GGX were heavily involved in experimental design, and also mainly involved in scientific correction of the draft manuscript. JX, YZ, and XZ were involved in sample collection and measurements of proteins, WNL, VLG, QW, YY, and RR, were involved in project discussion. All authors were involved in drafting the manuscript and revising it for critically important content. All authors have read and approved the final manuscript.

## Supplementary Material

Additional file 1: Figure S1 The effects of OT on MIA PaCa-2-2 cell proliferation using MTT assay. OT could cause the inhibition of cell growth and induce cell apoptosis on MIA PaCa-2 cell in a dose-dependent manner. IC50 of OT for MIA PaCa-2-2 cells was 14.95 μM. Click here for file
