# Supplementary figures and images for: Inhibition of transketolase by oxythiamine altered dynamics of protein signals in pancreatic cancer cells
Source: Exp Hematol Oncol. 2013 Jul 27;2:18. doi: 10.1186/2162-3619-2-18 (PMC3733980; doi:10.1186/2162-3619-2-18)

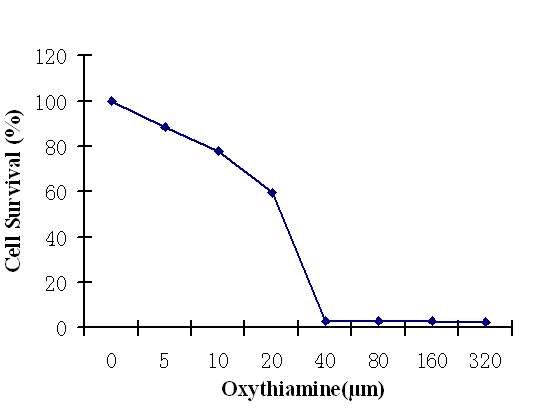

Supplement: Additional file 1: Figure S1 — The effects of OT on MIA PaCa-2-2 cell proliferation using MTT assay. OT could cause the inhibition of cell growth and induce cell apoptosis on MIA PaCa-2 cell in a dose-dependent manner. IC50 of OT for MIA PaCa-2-2 cells was 14.95 μM. [file 2162-3619-2-18-S1.jpeg]
